# P-334. Comparing Observed with Self-Reported Hand Hygiene Adherence Among Bedside Nurses at Two U.S. Hospitals

**DOI:** 10.1093/ofid/ofae631.537

**Published:** 2025-01-29

**Authors:** Prathit A Kulkarni, M Todd Greene, Sanjay Saint, Karen E Fowler, Serene Jacob, Laura Dillon, Michelle Espiritu, Nathan Houchens, Barbara Trautner

**Affiliations:** Michael E. DeBakey VA Medical Center / Baylor College of Medicine, Houston, Texas; University of Michigan and the Ann Arbor VA Healthcare System, Ann Arbor, MI; VA Ann Arbor Healthcare System, University of Michigan Medical School, Ann Arbor, Michigan; University of Michigan and the Ann Arbor VA Healthcare System, Ann Arbor, MI; VA Ann Arbor Healthcare System, Ann Arbor, Michigan; Michael E. DeBakey VA Medical Center / Baylor College of Medicine, Houston, Texas; Michael E. DeBakey VA Medical Center / Baylor College of Medicine, Houston, Texas; VA Ann Arbor Healthcare System / University of Michigan Medical School, Ann Arbor, Michigan; Michael E. DeBakey Veterans Affairs Medical Center / Baylor College of Medicine, Houston, Texas

## Abstract

**Background:**

Hand hygiene is recommended for all clinicians at entry to and exit from patients’ rooms. Adhering to such recommendations remains suboptimal possibly due, in part, to cognitive biases among clinicians who believe they are more compliant with hand hygiene than they actually are. We observed actual adherence to hand hygiene at room entry and exit among bedside nurses and compared these observations to self-reported perceptions of hand hygiene adherence.

**Methods:**

Hand hygiene observations of bedside nurses (during day and night shifts) were made during their normal workflow in six inpatient general medicine wards at two Department of Veterans Affairs academic medical centers in 2022. The frequency of hand hygiene being performed—with either alcohol-based hand rub or soap and water—was noted at entry to and exit from patient rooms. Nurses were also surveyed regarding how often they perceive they typically perform hand hygiene.

**Results:**

The participating units employed approximately 160 nurses. Hand hygiene was performed in 1,397 out of 3,690 (37.9%) room entry and in 2,159 out of 4,012 (53.8%) room exit opportunities, for a total of 3,556 out of 7,702 opportunities (46%). Hand hygiene was performed for a mean duration of 8.3 seconds at room exit. Among 92 survey respondents, approximately one-third had been a nurse for more than 20 years, 91% were female, 47% were Asian, and 8% were Hispanic. Survey respondents reported that they believe they perform hand hygiene in 95.1% of situations in which it is recommended.

**Conclusion:**

Our study reveals a stark disconnect between actual and perceived adherence to hand hygiene recommendations among bedside nurses in general medicine wards at two geographically distinct academic medical centers. Additionally, the mean duration for which hand hygiene was performed was less than the recommended duration of 20 seconds. Possible explanations for these findings include: social desirability bias and overestimation bias when answering survey questions; the need for multiple trips into and out of patients’ rooms to access medications or supplies located outside the room; and time pressures. Future approaches to improving hand hygiene adherence should ideally address cognitive biases among clinicians.

**Disclosures:**

**Barbara Trautner, MD, PhD**, Abbott Laboratories: Stocks/Bonds (Public Company)|AbbVie: Stocks/Bonds (Public Company)|Bristol Myers Squibb: Stocks/Bonds (Public Company)|Pfizer: Stocks/Bonds (Public Company)|Phiogen Pharma: Advisor/Consultant

